# Effects of Ridge and Furrow Planting Patterns on Crop Yield and Grain Quality in Dryland Maize–Wheat Double Cropping System

**DOI:** 10.3390/plants14193030

**Published:** 2025-09-30

**Authors:** Qihui Zhou, Ming Huang, Chuan Hu, Aohan Liu, Shiyan Dong, Kaiming Ren, Wenzhong Tian, Junhong Li, Fang Li, Guozhan Fu, Jinzhi Wu, Youjun Li

**Affiliations:** 1 Agricultural College, Henan University of Science and Technology, Luoyang 471023, China; zhouqihui@stu.haust.edu.cn (Q.Z.); huangming@haust.edu.cn (M.H.); hc23@stu.haust.edu.cn (C.H.); 230320170987@stu.haust.edu.cn (A.L.); dongshiyan@stu.haust.edu.cn (S.D.); renkaiming1848@163.com (K.R.); gzfu@haust.edu.cn (G.F.); 2 Luoyang Academy of Agriculture and Forestry Sciences, Luoyang 471023, China; lynlkxytwz@163.com (W.T.); lysnky@126.com (J.L.); lifanglifang123123@163.com (F.L.); 3 Luoyang Dryland Agriculture Test Site, Chinese Academy of Agricultural Sciences, Luoyang 471023, China

**Keywords:** ridge and furrow planting, dryland, maize–wheat double-cropping system, grain yield, quality

## Abstract

Ridge and furrow planting is a prevalent drought-resistant cultivation technique in dryland regions. Notably, the effects of this technology on crop grain yield and quality in dryland maize–wheat double-cropping systems remain limited. This study utilized a long-term positioning experiment initiated in 2004, which included five treatments: a permanent ridge and furrow with a border ridge of 133 cm row space (PRFBR); a ridge and furrow created each year with a border ridge of 133 cm row space (EYRFBR); a permanent ridge with a normal ridge of 100 cm row space (PRFNR); a ridge and furrow created each year with a normal ridge of 100 cm row space (EYRFNR), and a conventional flat planting pattern according to the local farmer (CF). The crop grain yield in 2015–2021, as well as the protein and phosphorus (P) and potassium (K) content in maize and wheat grains, and the protein components in winter wheat grains in 2020–2021 were investigated. The results showed that, compared to CF, all four ridge and furrow planting patterns significantly enhanced crop yield in dry and normal years, and the effects varied depending on crop species, with increases of 45.3–97.8% for wheat and 11.0–33.8% increases annually in dry years; and 24.5–51.6% increases for maize and 12.2–37.5% increases annually in the normal years. EYRFBR treatment increased wheat grain P and K content by 24.3% and 13.7%, as well as increasing the total protein, albumin, gliadin, soluble protein, and storage protein content by 9.7%, 22.3%, 9.6%, 14.5%, and 5.6%, whereas PRFNR reduced the glutenin content and glutenin/gliadin ratio in winter wheat grains by 5.1% and 10.9%, respectively. The yield achieved with a permanent ridge and furrow (PRF) surpassed that achieved when the ridge and furrow was created anew each year (EYRF), yet the normal ridge width (NR) outperformed the border ridge width (BR). However, the P, K, protein, and protein component content in wheat grains under EYRF was superior to that under PRF. Comprehensive evaluations through principal component analysis (PCA) and TOPSIS analysis consistently demonstrated that the EYRFBR treatment delivered optimal performance in yield and quality for winter and annual, while PRFNR achieved superior yield for summer maize. Consequently, in dryland maize–wheat double-cropping systems, an EYRFBR planting pattern should be recommended for high-yield and high-quality wheat production; however, the PRFNR planting pattern is more suitable for summer maize production.

## 1. Introduction

Maize (*Zea mays* L.) and wheat (*Triticum aestivum* L.) collectively account for over 60% of global cereal production [1,2]. Amid continuous population growth, accelerated economic development, and the increasing demand for high-quality cereals [3,4], enhancing both their yield and quality is of significance in ensuring national food security and improving human dietary health [5]. The summer maize–winter wheat (hereafter referred to as maize–wheat) rotation is one of the most popular and successful cropping systems in Northern China, producing about 30% and 45% of the national maize and wheat, respectively [6]. Although this system can efficiently utilize light and thermal resources, it suffers from scarce and unevenly distributed rainfall, poor soil fertility, and relatively underdeveloped cultivation practices [7], resulting in generally low and unstable crop yield and quality, particularly in rain-fed drylands [7,8]. Consequently, it is of great significance to develop agronomic practices to enhance both crop yield and quality in the maize–wheat double-cropping system in the drylands of China.

The ridge and furrow planting pattern is an excellent drought-resistant cultivation practice that combines ridge, furrow, and surface mulching [9], which has made great contributions to maize and wheat production in China [10,11]. For example, in Gansu Province, there are plans to implement a ridge and furrow planting pattern on 719,000 hectares of maize farmland [12]. In Shandong Province, the cumulative adoption of ridge and furrow planting for wheat has reached 18,000 hectares, resulting in a yield increase of 17.16 million kilograms and enhanced socio-economic benefits worth USD 21.5 million [13]. This technology efficiently modifies field topography, collecting rainwater [10], improving soil environmental conditions [11], and optimizing crop root morphology [14], thereby enhancing crop yield [15]. Research conducted on China’s Loess Plateau demonstrated that, compared to conventional flat planting, a ridge and furrow planting pattern increased maize yield by 39.3% [11], while enhancing the wheat yield by 51.6–115.2% [16]. Similarly, studies in Kenya showed that a ridge and furrow planting pattern with plastic mulching improved maize yields by 64.8–77.0% [17] and wheat yields by 18.2–90.3% [18] relative to flat planting. Notably, previous studies have indicated that the yield-enhancing effect of rainwater harvesting in the ridge and furrow planting pattern is closely associated with the precipitation level during crop growth [19,20]. Zhang et al. [20] stated that, under a rainwater harvesting system, the optimal yield gain was observed when the precipitation was in the range of 300–600 mm but declined when the rainfall was >600 mm. Wang et al. [21] found that under straw-covered ridge and furrow planting patterns, the optimal performance of maize yield occurred when seasonal rainfall was below 480 mm. Additionally, Li et al. [15] observed that a ridge and furrow planting pattern exhibited superior yield enhancement in the dry growing season. Yang et al. [22] also demonstrated that in dry and normal years, different ridge and furrow planting patterns under plastic film mulching resulted in greater differences in water harvesting and moisture conservation. These studies indicated that the effectiveness of a ridge and furrow planting pattern on crop yield varied under different precipitation types. Furthermore, in a maize–wheat rotation system, previous research has demonstrated that the ridge and furrow planting pattern is also effective for enhancing crop yields [23]. However, in the dryland maize–wheat double-cropping system, the effects of different ridge and furrow planting patterns on crop production under different precipitation types are still poorly understood.

Ridge and furrow planting technology also modifies the field microclimate and optimizes soil conditions, which generates beneficial ecological effects and creates favorable conditions for crop quality improvement [20,24]. Li et al. [25] demonstrated that the ridge and furrow planting pattern with ditch buried straw returned increased nitrogen (N) content and crude protein in maize grain. In another study [26], the ridge and furrow planting pattern significantly increased both oil and starch content in summer maize grains, but it had no significant effect on protein content and was associated with a general reduction in amino acid content. For winter wheat, Sun et al. [27] proposed that the ridge and furrow planting pattern enhanced both N assimilate supply and N assimilation capacity during the grain-filling stage, thereby increasing the grain protein content. However, Wang et al. [26] argued that a ridge and furrow planting pattern increased wheat grain starch content while decreasing protein content compared to that achieved with flat planting. Moreover, the ridge and furrow planting pattern also significantly increased the grain test weight and flour extraction rate, while it showed no statistically significant effects on flour water absorption and dough development time [28]. However, the effects of the ridge and furrow planting pattern on crop quality in the maize–wheat double-cropping system are still limited.

The agronomic effects of ridge and furrow planting are closely associated with ridging patterns and ridge width [9]. Previous studies have demonstrated that adopting no-tillage practices before establishing a ridge and furrow can increase net economic returns and topsoil nutrient content, but may also lead to the degradation of subsoil properties [29,30]. In contrast, preparing soil to an appropriate depth before ridging may improve subsoil properties and enhance crop nutrient acquisition [30,31]. Additionally, a field study [18] in the eastern African Plateau reported that appropriately increasing ridge width can make better use of light rainfall by enhancing its infiltration into the soil, increase rainwater collection efficiency, and improve soil water conservation. However, another study suggested that excessively wide ridges impeded water’s diversion into furrows while increasing volumetric soil water content in the ridge [32]. Liu et al. [19] also found that the soil water storage and the rate and duration of dry matter accumulation increased with increasing ridge width, but an excessively wide ridge did not significantly increase soil water storage, and ultimately reduced the effective duration of dry matter accumulation, decreasing yield and resource use efficiency in wheat. These results indicate that different ridge and furrow planting patterns exert different effects on crop performance. However, systematic investigations into the crop quality-regulating effects of these ridge and furrow planting patterns remain limited.

In summary, ridge and furrow planting patterns affected both crop yield and quality. However, previous studies mainly focused on mono-cropping with a plastic-mulched ridge and furrow sowing system in dryland regions or a ridge-planting system in irrigation areas. Notably, few studies have been carried out to explain how ridge and furrow planting patterns affect crop grain yield and quality in a dryland maize–wheat double-cropping system. Therefore, this study was conducted based on a 17-year (2004–2021) positioning experiment in a typical dryland maize–wheat double-cropping area, where maize was planted in furrows and wheat was planted on ridges and comprehensively accounted for ridging patterns and ridge width. We systematically investigated crop yield, grain protein content and protein yield, the grain phosphorus (P) and potassium (K) content of maize and wheat, and the characteristics of protein components in wheat grains. The objectives of the present study were to (1) determine the effects of different ridge and furrow planting patterns on crop yield and their response to different precipitation types, (2) evaluate the impacts of different ridge and furrow planting patterns on crop quality, and (3) provide theoretical and technical guidance for achieving high yield and high quality in a dryland maize–wheat double-cropping system.

## 2. Results

### 2.1. Grain Yield

Table 1 showed that precipitation type has a highly significant effect on crop yield, but its interaction effect with different ridge and furrow treatments was not significant. The yield of summer maize, winter wheat and the overall annual yield were all in the order of rainy year > normal year > dry year. For summer maize, the yield in the rainy year was significantly higher than in the normal and dry years (by 39.6% and 83.2%, respectively); meanwhile, the yield in the normal year was 31.2% higher than that in the dry year. For winter wheat, there was no significant difference in yield between the rainy and the normal year. However, the yield in both the rainy and the normal years was significantly higher than that in the dry year, with increases of 55.9% and 50.7%, respectively. Finally, regarding annual yield, the rainy and normal year yield was 26.4% and 18.8%—significantly higher than that in the dry year, respectively.

**Figure 1:**
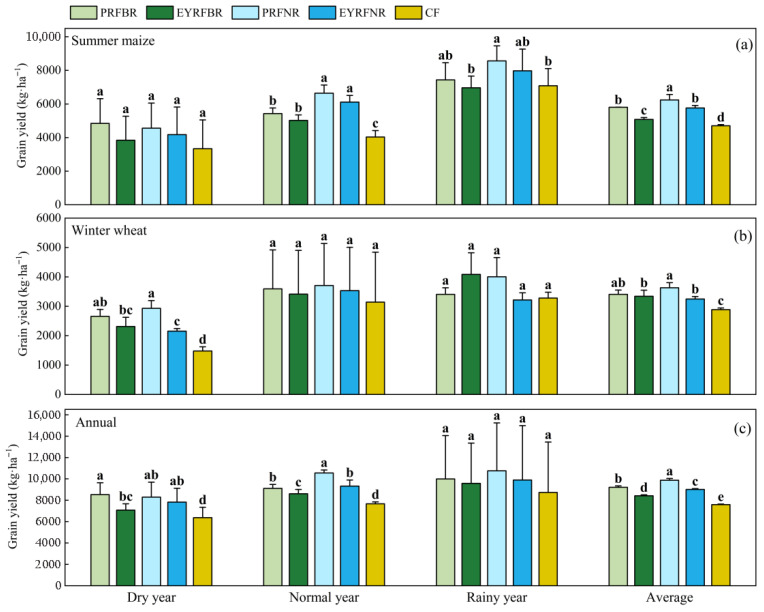
The effects of ridge and furrow planting patterns on grain yield of summer maize (**a**), winter wheat (**b**) and annual year (**c**) in dryland summer maize–winter wheat double-cropping system under different precipitation types. Note: PRFBR represents permanent ridge and furrow and border ridge of 133 cm row space; EYRFBR represents a ridge and furrow created each year with normal ridge of 133 cm row space; PRFNR represents a permanent ridge and furrow and a normal ridge of 100 cm row space; EYRFNR represents a ridge and furrow created each year with a normal ridge of 100 cm row space; CF represents a conventional flat planting pattern according to the local farmer. The error line represents the standard deviation. Different lowercase letters indicate that the difference among treatments within the same precipitation type is significant at *p* < 0.05.

Figure 1 illustrated that all ridge and furrow planting patterns were beneficial for improving the crop yield compared to the conventional flat planting pattern (CF). The effectiveness was regulated by ridge-raising patterns and ridge width. On the whole, there was a rule that establishing a permanent ridge and furrow (PRF) was better than creating a fresh ridge and furrow each year (EYRF), and a normal ridge width of 100 cm (NR) was better than a border ridge width of 133 cm (BR). For summer maize, in a normal year, the yield of a permanent ridge and furrow with a border ridge with 133 cm row space (PRFBR), that of a fresh ridge and furrow created anew each year with a normal ridge of 133 cm row space (EYRFBR), that of a permanent ridge and furrow with a normal ridge of 100 cm row space (PRFNR), and that of a ridge and furrow newly created each year with a normal ridge of 100 cm row space (EYRFNR) significantly increased by 34.6%, 24.5%, 64.8%, and 51.6%, respectively, compared to CF. NR significantly increased yield by 22.4% and 21.8% under PRF and EYR, respectively, compared to BR. In the rainy year, PRFNR treatment significantly increased by 20.9% compared to CF.

For winter wheat, in a dry year, PRFBR, EYRFBR, PRFNR, and EYRFNR significantly increased yield by 79.6%, 56.1%, 97.8%, and 45.3%, respectively, compared to CF. Among them, PRF increased yield by 15.0% (*p* > 0.05) and 36.1% (*p* < 0.05) under BR and NR, respectively, compared to EYRF. However, in the normal and rainy year, there were no significant differences among the five treatments.

In terms of annual yield, compared to CF, PRFBR, EYRFBR, PRFNR, and EYRFNR significantly increased by 33.8%, 11.0%, 30.2%, and 22.8% in the dry year, as well as 18.8%, 12.2%, 37.5%, and 21.5% in the normal year, respectively. The effects of ridge patterns on annual yield varied with precipitation type. Compared to EYRF, PRF increased the yield by 15.9% (*p* < 0.05) and 5.9% (*p* > 0.05), respectively under BR and NR in the dry year, and in a normal year, it significantly increased by 5.8% and 13.2%, respectively. Furthermore, NR significantly outperformed BR in yield in the normal year. However, in the rainy year, there was no significant difference in annual yield among treatments.

### 2.2. Protein Content and Protein Yield

**Figure 2:**
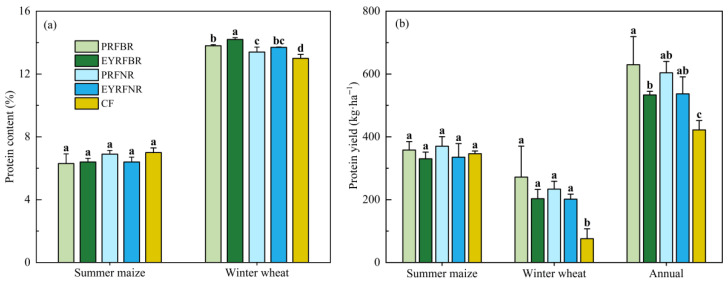
Effects of ridge and furrow planting patterns on protein content (**a**) and protein yield (**b**) in maize and wheat in a dryland summer maize–winter wheat double-cropping system in 2020–2021. Note: PRFBR represents a permanent ridge and furrow and a border ridge of 133 cm row space; EYRFBR represents a ridge and furrow created anew each year with a normal ridge of 133 cm row space; PRFNR represents a permanent ridge and furrow and a normal ridge of 100 cm row space; EYRFNR represents a ridge and furrow created anew each year with a normal ridge of 100 cm row space; CF represents a conventional flat planting pattern according to the local farmer. Different lowercase letters indicate that the difference among treatments is significant at *p* < 0.05.

Ridge and furrow planting patterns had no significant effect on the grain protein content and protein yield of summer maize, but they significantly improved those of winter wheat, and the effect of protein content was regulated by ridge raising patterns and ridge width (Figure 2). Compared to CF, PRFBR, EYRFBR, PRFNR, and EYRFNR significantly increased winter wheat grain protein content by 6.6%, 9.7%, 3.6%, and 5.4%, respectively, in which EYRF was 3.0% (*p* < 0.05) under BR and 1.7% (*p* > 0.05) better under NR than PRF, respectively. BR was 2.8% (*p* < 0.05) under PRF and 4.1% (*p* < 0.05) better under EYRF than NR, respectively. Furthermore, compared to CF, the protein yield of winter wheat significantly increased by 258.6%, 168.1%, 208.2%, and 166.0% under PRFBR, EYRFBR, PRFNR, and EYRFNR, respectively. The annual protein yield was also increased by 49.2%, 26.4%, 43.1%, and 27.2%, respectively. In general, the ridge and furrow planting pattern significantly increased the crop protein yield of the maize–wheat double-cropping system in drylands, especially for wheat protein yield.

### 2.3. P and K Contents in Crop Grains

**Figure 3:**
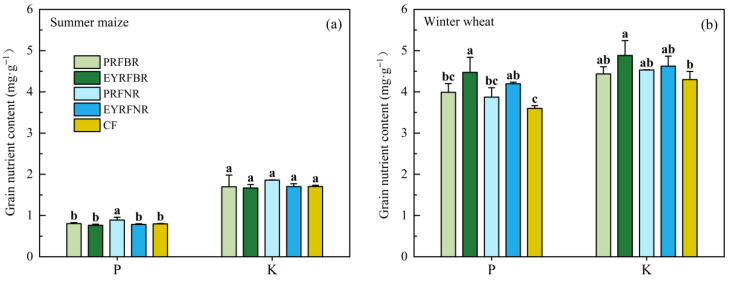
Effects of ridge and furrow planting patterns on the phosphorus and potassium content in summer maize (**a**) and winter wheat (**b**) grains in dryland summer maize–winter wheat double-cropping system in 2020–2021. Note: PRFBR represents a permanent ridge and furrow and a border ridge with 133 cm row space; EYRFBR represents a ridge and furrow created each year with a normal ridge with 133 cm row space; PRFNR represents a permanent ridge and furrow and a normal ridge with 100 cm row space; EYRFNR represents a ridge and furrow created each year with a normal ridge of 100 cm row space; CF represents a conventional flat planting pattern according to the local farmer. The error line represents the standard deviation. Different lowercase letters indicate that the difference among treatments is significant at *p* < 0.05.

The effects of ridge and furrow planting patterns on grain P and K content also varied with the crop species (Figure 3). For summer maize, there were no significant differences in grain P and K content among treatments except that the P content under PRFNR was higher than that under other treatments. For winter wheat, ridge and furrow planting patterns generally exhibited a trend of increasing grain P and K content. Compared to CF, EYRFBR significantly increased grain P and K content by 24.3% and 13.7%, respectively; and EYRFNR significantly increased grain K content by 16.6%. Furthermore, the content of P and K in wheat grains under EYRF was better than that under PRF; thus, the EYRFBR treatment had the highest values. In addition, the increase in P content induced by the ridge and furrow planting pattern was greater than the K content.

### 2.4. Contents of Protein Components in Winter Wheat

**Figure 4:**
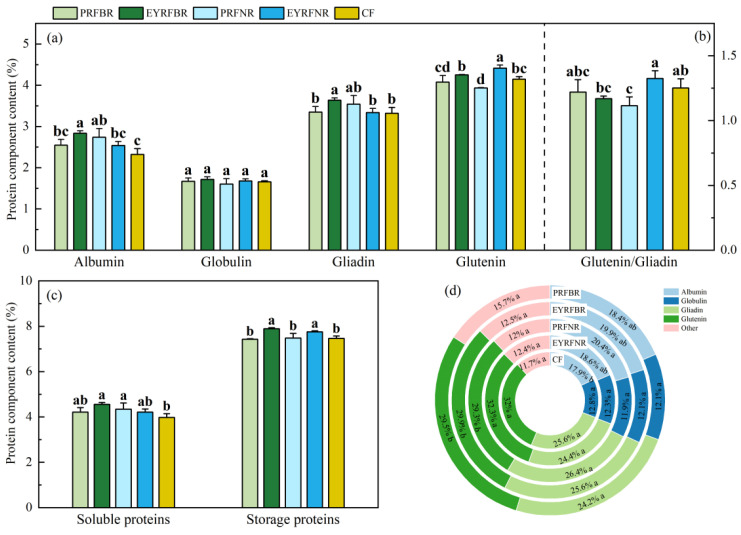
The effects of ridge and furrow planting patterns on the content (**a**,**c**) and the ratios (**b**,**d**) of protein components of winter wheat in a dryland summer maize–winter wheat double-cropping system in 2020–2021. Note: PRFBR represents permanent ridge and furrow and border ridge of 133 cm row space; EYRFBR represents ridge and furrow created each year with normal ridge of 133 cm row space; PRFNR represents permanent ridge and furrow and normal ridge of 100 cm row space; EYRFNR represents ridge and furrow created each year with normal ridge of 100 cm row space; CF represents conventional flat planting pattern according to the local farmer. The error line represents the standard deviation. Different lowercase letters indicate the difference among treatments is significant at *p* < 0.05.

Figure 4 showed that the albumin, globulin, gliadin, soluble protein and storage protein content in winter wheat grains were all highest under the EYRFBR treatment. Compared to CF, EYRFBR and PRFBR significantly increased the albumin content by 22.3% and 18.1% and the soluble protein content by 14.5% and 9.2%, respectively, and EYRFBR significantly increased gliadin content by 9.6%. Compared to CF, the grain glutenin content of winter wheat under NR was significantly different; EYRFNR significantly increased the grain glutenin content by 6.5%, while PRFNR significantly decreased it by 5.1%. Compared to PRF, EYRF significantly improved the glutenin content by 4.3% and 12.2% under BR and NR, respectively. The storage protein content was also regulated by ridge and furrow planting patterns. Compared to CF, EYRFBR and EYRFNR significantly increased the storage protein content by 5.6% and 3.8%, respectively. EYRF significantly increased storage protein content by 6.2% and 3.7% under BR and NR compared to PRF, respectively. PRFNR significantly decreased the glutenin/gliadin ratio by 10.9% compared to CF. In addition, the fluctuation range of the glutenin content and glutenin/gliadin ratio in winter wheat grains under NR was greater than that under BR.

From the view of the proportion of protein components to total protein in winter wheat grains (Figure 4d), compared to CF, ridge and furrow planting patterns increased the albumin proportion while reducing the glutenin proportion to a certain extent, except EYRFNR showed minimal changes.

### 2.5. Comprehensive Evaluation

#### 2.5.1. Principal Component Analysis

**Figure 5:**
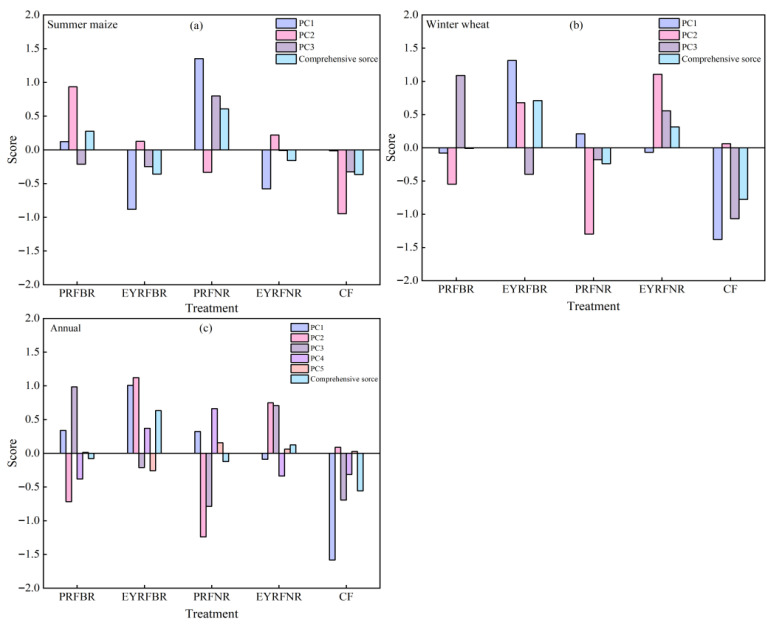
Principal component score of grain yield and quality for summer maize (**a**), winter wheat (**b**) and annual year (**c**) in dryland summer maize–winter wheat double-cropping system under different ridge and furrow planting patterns. Note: PRFBR represents a permanent ridge and furrow and border ridge of 133 cm row space; EYRFBR represents a ridge and furrow created each year with a normal ridge with 133 cm of row space; PRFNR represents a permanent ridge and furrow and a normal ridge with 100 cm of row space; EYRFNR represents a ridge and furrow created each year with a normal ridge with 100 cm row space; CF represents a conventional flat planting pattern according to the local farmer.

Through principal component analysis, the multiple interrelated single indicators of summer maize, winter wheat and annual yield, and quality parameters in 2020–2021 were transformed into few independent comprehensive indicators (Supplementary Table S1, Figure 5). The principal components were extracted by principal component eigenvalue greater than 1. Three, three, and five principal components were respectively obtained for summer maize, winter wheat, and annual perspective, with an accumulative contribution rate up to 91.9%, 84.5%, and 88.5%. The comprehensive scores (Figure 5) were as follows: PRFNR > PRFBR > EYRFNR > EYRFBR > CF for summer maize, and EYRFBR > EYRFNR > PRFBR > PRFNR > CF for winter wheat and annual.

#### 2.5.2. TOPSIS Analysis

**Figure 6:**
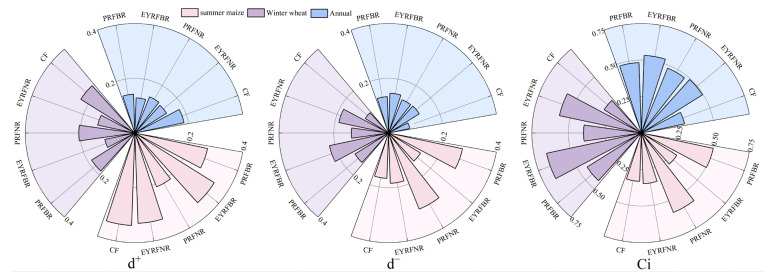
TOPSIS analysis of grain yield and quality in a dryland summer maize–winter wheat double-cropping system under different ridge and furrow planting patterns. Note: di^+^: The distance of each evaluation scheme from the positive ideal solution; di^−^: The distance of each evaluation scheme from the negative ideal solution; Ci: relative closeness coefficient. PRFBR represents a permanent ridge and furrow and a border ridge with 133 cm of row space; EYRFBR represents a ridge and furrow created each year with normal ridge with 133 cm of row space; PRFNR represents a permanent ridge and furrow and normal ridge with 100 cm of row space; EYRFNR represents a ridge and furrow created each year with a normal ridge with 100 cm of row space; CF represents a conventional flat planting pattern according to the local farmer.

Further TOPSIS analysis (Figure 6) showed that the closeness coefficients (Ci) of summer maize were ranked as PRFNR (0.585) > PRFBR (0.503) > EYRFNR (0.347) > CF (0.330) > EYRFBR (0.286). The Ci of different treatments for both winter wheat and annual fallowed the same order: EYRFBR > EYRFNR > PRFBR > PRFNR > CF, with values of 0.664, 0.574, 0.438, 0.396, and 0.301 for winter wheat, and 0.532, 0.494, 0.483, 0.479, and 0.302 for annual perspective, respectively. These results suggest that PRFNR for summer maize and EYRFBR for winter wheat and annual production better align with the goals of high yield and high quality in the dryland maize–wheat double-cropping system.

## 3. Discussion

### 3.1. Effect of Ridge and Furrow Planting Patterns on Crop Yield in a Dryland Maize–Wheat Double-Cropping System and Its Response to Precipitation Type

Previous studies showed that ridge and furrow planting pattern can not only efficiently collect rainfall, promote water infiltration, and reduce ineffective evaporation of soil water [10], but can also optimize field environmental conditions [11], thereby increasing crop yield. In the present study, all four ridge and furrow planting patterns combined with straw mulching improved crop yield compared to CF, which is consistent with previous results [11,16,17,18]. However, our study further revealed that the effects of ridge and furrow planting pattern on crop yield were significantly influenced by both crop and precipitation types (Figure 1). For summer maize, compared to CF, the yields of the ridge and furrow planting pattern were significantly increased only in the normal year, and the increase under NR was higher than that under BR. In dry years, although the yields of summer maize under the ridge and furrow planting pattern were also higher than CF, the differences were not significant. The major reason for this may be that the rainfall in the study area is mainly distributed in the summer season, along with the summer maize growth stage. Thus, the low rainfall in the dry year reduced the efficacy of water harvesting induced by the ridge and furrow planting pattern, and its effect on improving the summer maize yield by water harvesting was limited [33]. Furthermore, in the rainy year, all treatments maintained a higher level of soil moisture, and there were also no significant yield differences among treatments except for PRFNR. The conclusions drawn from simulated rainfall experiments further substantiate this view [33,34]. They found that when seasonal rainfall reached 400 mm, summer maize yield under ridge and furrow planting patterns showed no further significant increase.

Thus, the ridge and furrow planting pattern only fully demonstrated its optimal water conservation and yield enhancement effects for summer maize in the normal year. We also found that, as the ridge width increased from 60 ± 5 cm to 95 ± 5 cm, a significant reduction (21.8–22.4%) in summer maize yield was observed. This reduction was primarily attributed to the combined effects of increased row spacing (from 100 cm to 133 cm) and decreased plant spacing (from 22.5 cm to 16.7 cm), which consequently constrained root growth and distribution, affected canopy structure, and reduced the canopy light interception rate [35]. Ding et al. [36] demonstrated that under the same planting density, increasing the row spacing from 80 cm to 100 cm resulted in 34.5–51.8% and 35.2–54.5% higher light transmittance at the silking and milking stages, respectively. However, this change concurrently reduced the extinction coefficient by 6.8–14.3% and 8.3–13.3% during these stages, consequently decreasing radiation use efficiency and ultimately leading to yield reduction. Similarly in our study, when the row spacing was increased from 100 cm to 133 cm, potential light leakage between the two rows and reduction in radiation use efficiency were observed [37], ultimately reducing per-plant productivity and grain yield. Additionally, it is worth noting that another reason may be that overly wide ridges are inefficient at diverting water into furrows, thereby reducing the efficiency of ridge–furrow rainwater harvesting [31].

Our findings indicated that compared to CF, all the ridge and furrow planting patterns significantly increased wheat yield only in the dry year, while no significant differences were observed in the normal or rainy years. This aligns with the findings of Qiang et al. [16] from their study in Shanxi, China. The reason may be that under sufficient rainfall conditions, the main function of ridge and furrow planting pattern for collecting rainfall was lost, and consequently exhibited no significant effects on wheat growth [33]. Ali et al. [38,39,40] also reported that when simulated rainfall during the wheat growing season reached 275 mm, the ridge and furrow planting pattern showed no significant effects on wheat’s photosynthetic characteristics, yield, water use efficiency, grain weight, and grain filling rates. Moreover, our study also found that PRF outperformed EYRF in terms of wheat yield. This was mainly ascribed to the permanent ridge and furrow pattern maintained under long-term no-till conditions, which promoted the formation of soil porosity and water-stable aggregates [41,42], thereby enhancing water retention and soil moisture conservation. Furthermore, this pattern also improved nutrient content [41] and enzyme activities [42] in the topsoil layer, thus creating favorable soil conditions for wheat yield improvement.

It is worth mentioning that differential responses to precipitation types were observed between summer maize and winter wheat. Zhang et al. [20] also pointed out that the yield-increasing effect of rainwater harvesting during the crop growth stage was closely associated with the precipitation type. A study by Mhawej et al. [43] in the United States indicated that maize has higher evapotranspiration than wheat, and wheat shows a greater drought tolerance. In addition, maize, as a C4 plant, exhibits higher water use efficiency and greater water demand than the C3 species wheat [44]. This physiological distinction may explain why the ridge and furrow planting pattern exhibited a significant yield improvement effect on wheat in the dry year and for maize in the normal year in our study. Finally, the annual yields under ridge and furrow planting patterns were significantly higher than those under CF in both dry and normal years. This study provides further empirical evidence supporting previous studies, and ultimately concludes that the ridge and furrow planting pattern is suitable for improving yields in a dryland maize–wheat double-cropping system.

### 3.2. Effect of Ridge and Furrow Pattern on Crop Yield Quality in Dryland Maize–Wheat Double-Cropping System

The levels of nutrient content in grains not only reflect the growth status and intensity of nutrient uptake/utilization in crops, but also indicate their dietary nutritional value [3,45]. Different ridge and furrow planting patterns can influence crop growth and development by altering soil nutrient status, thereby affecting the nutritional quality of crop grains [46]. A study by Ma et al. [47] in Hebi, China, indicated that the ridge and furrow planting pattern increased the N, P, and K content in winter wheat grains by 15.9% (*p* < 0.05), 6.9%, and 2.1%, respectively, compared to flat planting. Our research yielded similar results; compared to CF, all four ridge and furrow planting patterns showed a trend toward increasing the nutrient content in winter wheat grains, with EYRFBR treatment exhibiting the most pronounced effects. This may be related to the favorable ecological environment created by the ridge and furrow planting pattern. Previous research has shown that the ridge and furrow planting pattern not only increases rainwater’s infiltration into deep soil [48], but also optimizes soil thermal regimes [11], increases the soil nutrient content, and enhances microbial and enzyme activity [9]. These combined effects create favorable conditions that can enhance nutrients’ uptake and utilization by crops, ultimately contributing to improved nutritional quality in cereal grains [24,31]. Additionally, the enhancement of nutrient content under a ridge and furrow planting pattern in our study was also associated with straw mulching, which has been widely demonstrated to increase soil properties and soil nutrients [49]. Meanwhile, for summer maize, compared to CF, the ridge and furrow planting patterns showed no significant differences with regard to grain P and K content. This differential response may be attributed to the optimized canopy architecture and improved photosynthetic characteristics of maize planted in the furrow, which promoted greater assimilates accumulation [50], thus diluting the effect of ridge and furrow planting patterns on nutrient accumulation [51], even resulting in a marginal decrease in grain protein content in summer maize. Furthermore, the nutrient indices for summer maize were measured during dry years (Table 2), and the water-harvesting capacity and nutrient uptake improvements of ridge and furrow planting pattern were limited [44,52], which may explain the different response.

Our study also further demonstrated that the P and K content in wheat grains was significantly regulated by the ridge raising pattern and ridge width. There was a rule that EYRF was better than PRF, and BR was better than NR, with particularly significant differences observed in the grain protein content. These results could be related not only to the dilution effect caused by higher yields [51], but also the differential environmental improvements under various ridge and furrow planting patterns [9]. Our previous findings [9] indicated that a permanent ridge and furrow planting pattern reduced bulk density and enhanced soil nutrient content. In addition, long-term no-till conditions under PRF promoted nutrient enrichment in the topsoil [29,30]. In contrast, the ridge and furrow created each year under EYRF significantly stimulated soil nutrient mineralization and effectively optimized deep soil nutrient content and enzyme activity [9,30], thereby promoting root development and plant nutrient utilization of wheat [31]. Consequently, EYRF exhibited superior nutrient content in winter wheat grains compared to PRF. A field study also reported that plowing before ridge and furrow planting pattern facilitated root proliferation and increased N accumulation, translocation and grain protein content compared to no-till practice, which was similar to the findings of our research [31]. On the other hand, different ridge widths changed the proportion of the ridges and furrows, resulting in different capacities for rainwater harvesting, soil moisture retention, and soil hydrothermal conditions [19,53]. In this study, BR exhibited higher grain nutrient content than NR. A possible reason for this may be that the appropriate increase in the ridge width under the BR pattern was conducive to enhancing soil temperature and water content in ridge [18,53], where winter wheat was planted. In addition, the reduction in the ridge width under NR may have been more conducive to the radiation capture and border-row effect of wheat [32]. All of these factors were beneficial for yield improvement, but diluted nutrients in grains to a certain extent [51].

The contents and ratios of protein composition in grains are closely related to wheat quality [54]. Studies have shown that soluble proteins (albumin and globulin) are the most important indicators of nutritional value [55], while storage proteins are important factors in determining the flour blending, dough properties, and end-use quality, and gliadin is generally considered responsible for dough extensibility, whereas glutenin is more important for dough elasticity or dough strength [56,57]. Furthermore, the glutein/gliadin ratio is an important indicator affecting the baking quality of bread and the quality of steamed buns [54]. In our study, compared to CF, ridge and furrow planting patterns increased winter wheat grain total protein content and most of the parameters of protein components while enhancing the grain yield, collectively contributing to a significant increase in winter wheat protein yield (Figure 4). In particular, EYRFBR resulted in the highest amounts of albumin, globulin, gliadin, soluble protein, and storage protein in winter wheat grains, which contributed to superior nutritional and processing quality. Sun et al. [27] found that the ridge and furrow planting pattern increased the free amino acid content in wheat flag leaves during the early grain-filling stage and enhanced nitrate reductase activity in the mid-to-late grain-filling stages, thereby increasing the grain protein content in wheat. Wu et al. [58] demonstrated that summer subsoiling combined with the ridge and furrow planting pattern enhanced both N uptake efficiency and internal efficiency in winter wheat, thus significantly increasing the content of grain total protein and its components. Additionally, previous studies have demonstrated that the pre-anthesis accumulation and re-mobilization of N were more directly used for grain soluble protein synthesis, while post-anthesis N uptake exerts greater influence on storage protein synthesis [54,59]. Ma et al. [47] reported that compared to flat planting, the ridge and furrow planting pattern increased wheat N uptake by 17.2%, consequently improving grain N content. Li et al. [25] demonstrated that compared to flat planting, the ridge and furrow planting pattern significantly enhanced grain protein content through improving above-ground and grain N accumulation by 27.3–31.2% and 40.0–92.3%, respectively. Zhang et al. [31] also demonstrated that plowing before the ridge and furrow planting pattern enhanced root growth during grain-filling increased root and above-ground N translocation by 26.0–45.8% and 18.3–24.6%, respectively. These studies may explain our results.

To the best of our knowledge, studies about the effects of ridge raising patterns and ridge width on grain protein components are limited. Our study revealed that EYRF exhibited significantly higher glutenin and storage protein content in winter wheat grains compared to PRF. Furthermore, we also found that the fluctuation range of glutenin content and the glutenin/gliadin ratio of winter wheat grain under NR was greater than that under BR. This may suggest that BR has more stable quality characteristics than NR. The reason for this may be that PRF patterns induced soil compaction and subsoil nutrient depletion, impairing sustained N uptake and potentially triggering post-anthesis N exhaustion [30]. In contrast, EYRF promoted deeper root proliferation, enabling continuous access to deep soil nutrients and maintaining post-anthesis N supply [31], which may result in significantly higher storage protein and glutenin content compared to PRF. The reason maybe also ascribed to plant N characteristics of wheat under different planting patterns. Regrettably, we did not assess the characteristics of plant N accumulation, translocation, and distribution, which could explain the changes in the above-mentioned quality parameters. Thus, the effects of the ridge and furrow pattern on plant N characteristics should be focused on in the future.

### 3.3. Comprehensive Evaluation of Ridge and Furrow Pattern on Crop Yield and Quality in Dryland Maize–Wheat Double-Cropping System

Both PCA and TOPSIS analysis enable comprehensive evaluation of complex traits in crops. Sun et al. [60] employed PCA to evaluate the comprehensive performance of spring maize under treatments combining organic manure with reduced chemical fertilizer applications. Through TOPSIS analysis, Hamani et al. [61] identified optimal irrigation and N fertilization regimes for winter wheat under drip-irrigation systems in the North China Plain in China. In our study, the integrated comprehensive evaluation combining yield and quality metrics under PCA (Supplementary Table S1, Figure 5) and TOPSIS (Figure 6) analysis consistently demonstrated that EYRFBR achieved optimal yield and quality performance for both winter wheat and from an annual perspective. Therefore, we concluded that the EYRFBR treatment represents the optimal ridge and furrow planting pattern for achieving both a high yield and superior quality in a dryland maize–wheat double-cropping system. Meanwhile, for summer maize, the two analyses consistently indicated that the PRFNR treatment delivered the optimal performance for summer maize. From the perspective of different ridge and furrow planting patterns, both PCA and TOPSIS analyses suggested that NR was superior to BR in terms of maize yield, while EYRF outperformed PRF with regard to winter wheat yield and quality. Therefore, when implementing ridge and furrow planting pattern in similar regions, excessively wide ridges are not recommended for maize cultivation due to suboptimal resource use efficiency and consequent yield reduction. For winter wheat, adopting plowing with frequent ridging operations is advised to enhance grain yield and quality.

Notably, this study was only conducted in the southern Loess Plateau, focusing on two ridge-forming patterns and two ridge width arrangements. Our research did not involve the formation mechanisms of yield and quality. Further research is needed to clarify the efficacy of this technology under different ecological, soil, and rainfall conditions, as well as the cultivation techniques compatible with the ridge and furrow planting pattern in a dryland maize–wheat double-cropping system.

## 4. Materials and Methods

### 4.1. Experimental Site Description

**Figure 7:**
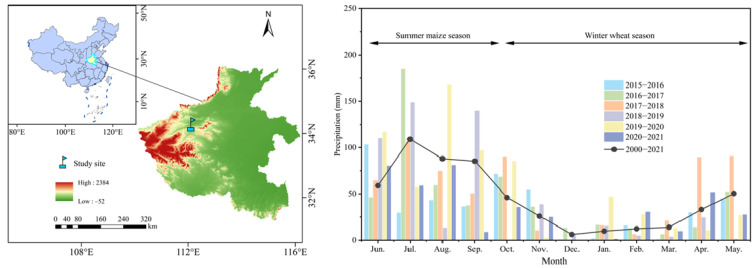
Location of the test site and monthly precipitation during June 2015 to May 2021.

The present experiment was performed from June 2004 to June 2021 at the Luoyang Dryland Agriculture Test Site, Chinese Academy of Agricultural Sciences, Henan Province, China (34°37′12″ N, 112°27′36″ E) (Figure 7). The experimental site is located in a drought-prone region at the southeast edge of the Loess Plateau, where the altitude is 130 m and has a temperate continental monsoon climate. The average annual temperature is 14.6 °C, and average annual precipitation of 549 mm. Maize–wheat rotation is the main local cropping system. Soils at the experimental site are classified as yellow-brown earthy cinnamon. The bulk density of the cultivated layer was 1.5 g m^−3^. At the initiation of the experiment in 2004, the basic properties in the 0–20 cm soil layer were as follows: organic matter content of 15.6 g kg^−1^, total N content of 0.9 g kg^−1^, alkali-hydrolysable N content of 62.5 mg kg^−1^, available phosphorus content of 10.4 mg kg^−1^, and available potassium content of 166.0 mg kg^−1^. The monthly precipitation from 2015 to 2021 and the average from 2001 to 2021 are shown in Figure 7. The precipitation types for each year were classified according to the dryness index (DI) [62], as detailed in Table 2.

### 4.2. Experimental Design and Field Management

**Figure 8:**
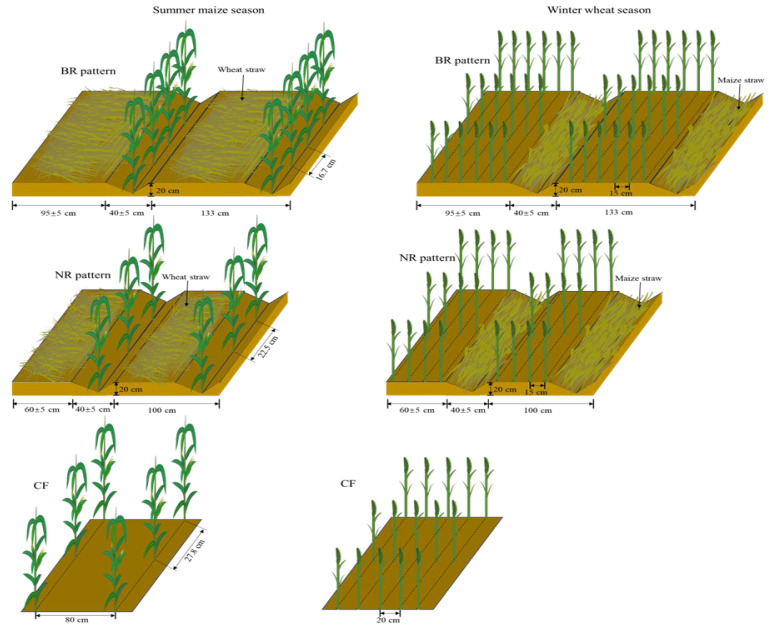
Planting diagram.

This study applied a two-factor experimental treatment in addition to the control. The treatments comprised two ridge and furrow patterns: (i) a permanent ridge and furrow (PRF) and (ii) a ridge and furrow created each year (EYRF) with two ridge widths, (i) a border ridge with 133 cm row space (BR) and (ii) a normal ridge with 100 cm row space (NR). A conventional flat planting pattern with 80 cm row space for maize and 20 cm row space for wheat according to the local farmer system (CF) was used as the control. Thus, the experiment comprised a total of five treatments: PRFBR, EYRFBR, PRFNR, EYRFNR, and CF. The details of the treatment are listed in Figure 8 and Table 3.

A pool planting methodology with three replicates for each treatment was adopted. Each plot was 16 m^2^ (4 m × 4 m) in size. The chemical fertilizers used in this study were urea (46% N) in the maize season and compound fertilizer (N:P_2_O_5_:K_2_O = 15:15:15) in the winter wheat season. In the summer maize season, the rates of 138 kg N ha^−1^ were manually top-dressed with rainfall around the jointing stage. In the wheat season, compound fertilizer was applied as the basal fertilizer with application rates of 112.5 kg N ha^−1^, 112.5 kg P_2_O_5_ ha^−1^, and 112.5 kg K_2_O ha^−1^. For the treatments with tillage, fertilizers were uniformly broadcast by hand before plowing. For treatments without tillage, fertilizers were manually band-applied between seed rows at 15–20 cm depth by imitating a no-tillage fertilization seeder. The experiment employed the maize cultivar “Luoyu 114” consistently from 2015 onwards, while the wheat cultivars used were “Luohan 7” (2015–2020) and “Luohan 22” (2020–2021). Summer maize was manually sowed in early June and harvested in late September, with a planting density of 45,000 plant ha^−1^. The row configurations varied depending on the ridge width: 133 cm row spacing for border patterns, 100 cm row spacing for normal ridge width patterns, and 80 cm for the conventional flat planting pattern. Winter wheat was manually sowed in early or mid-October and harvested in late May or early June of the following year, with a plant density of 270 plant m^−2^. During the experiment, there was no irrigation. Weeds, pests, and diseases were controlled with herbicides and pesticides according to local farming practices.

### 4.3. Measurements and Methods

#### 4.3.1. Grain Yield

Once the maize and wheat had matured, all plants in each plot were harvested manually. After air-drying, the plants in each plot were threshed, and the grain was weighed to determine the grain yield. Briefly, 50 ± 5 g grains were oven-dried at 80 °C to a constant weight, and the moisture of the grains was measured, and the grain yield (kg·ha^−1^) per hectare was calculated based on a grain moisture content of 14.0% for maize and 12.5% for wheat [7].

#### 4.3.2. Grain Protein Content and Protein Yield

In the experimental years 2020–2021, 50 g of maize and wheat grains were oven-dried and ground and then digested with H_2_SO_4_-H_2_O_2_ to determine the N content. The N content was determined using a high-resolution digital colorimeter auto analyzer 3 (AA3, SEAL Company, Norderstedt, Germany) [63]. The protein content and protein yield were calculated using the following equations:

Maize protein content (%) = N content in grains × 6.25 [64]

Wheat protein content (%) = N content in grains × 5.7 [65]

Protein yield (kg ha^−1^) = Grain dry weight × protein content (%) [8]

#### 4.3.3. Grain P and K Content

In the experimental year of 2020–2021, 50 g of maize and wheat grains were oven-dried and ground and then digested with H_2_SO_4_-H_2_O_2_ to determine the content of P and K. The P content was determined using a high-resolution digital colorimeter auto analyzer 3 (AA3, SEAL Company, Norderstedt, Germany), and the K content was determined using a flame photometer (M410, Sherwood, Cambridge, UK) The P and K content in wheat and maize grains is expressed on a dry weight basis [63].

#### 4.3.4. Grain Protein Components

In 2020–2021, the content of protein components in wheat grains was determined as previously described [54]. Here, 0.50 g of whole meal flour was precisely weighed and subjected to albumin extraction. This extraction process involved mixing the flour with 5 mL of pure water in a plastic centrifuge tube, followed by oscillation (20 min) and centrifugation (4000 r, 7 min). This procedure was repeated four times, and the resulting extract was collected as albumin. Similarly, the residue remaining in the tube underwent extraction with separate solutions to isolate the fractions of globulin, gliadin, and glutenin. Specifically, 2% NaCl, 75% ethanol, and 0.2% NaOH solutions were employed for the extraction process, respectively. The extraction protocol mirrored that described above and was repeated four times for each fraction. Subsequently, the concentration of each protein fraction was determined using the Kjeldahl method (H8750, Haineng company, Shanghai, China). Soluble proteins were quantified as the sum of albumin and globulin, while storage proteins comprised gliadin and glutenin. The glutenin/gliadin ratio (Glu/Gli) was calculated as the relative content ratio of glutenin to gliadin [59,66].

### 4.4. Calculation of Comprehensive Evaluation Value

Crop yield and quality represent different dimensions that are not amenable to direct assessment. Therefore, we adopted the following two methods to conduct a comprehensive evaluation of yield and quality.

#### 4.4.1. Principal Component Analysis (PCA)

We standardized the raw data, then calculated the correlation coefficient matrix and extracted principal components with eigenvalues greater than 1. The weight of principal components were calculated using the contribution rate of each comprehensive index, Then we calculated scores for each principal component and composite scores to conduct a comprehensive evaluation of crop yield and quality [60,67].

#### 4.4.2. Calculation of Comprehensive Evaluation Values for Each Scheme Using Technique for Order Preference by Similarity to Ideal Solution (TOPSIS)

The entropy weight method was used for objective weight analysis, and then we used the TOPSIS method to evaluate each scheme by measuring the distance from the ideal solution. The best scheme is the one that is closest to the optimal solution and furthest from the worst solution. The comprehensive evaluation value of each treatment is represented by Ci (0 < Ci < 1), where a value closer to 1 indicates that the scheme is more conducive to crop high-yield and high-quality [61].

### 4.5. Statistical Analysis

Data collation was carried out using Microsoft Excel 2019 software. Statistical methods were performed using one-way analysis of variance (ANOVA) to examine the grain yield, precipitation use efficiency, and quality indicators. The difference (Duncan, at a 0.05 probability level) test was subsequently applied in SPSS software (version 27, IBM, New York, NY, USA) to determine significant differences among the treatments, and principal component analysis (PCA) and TOPSIS (Technique for Order Preference by Similarity to Ideal Solution) analysis was conducted later. The graphical presentation was generated using Origin software (version 2022, Origin Lab Corporation, Northampton, MA, USA).

## 5. Conclusions

Compared to conventional flat planting, the ridge and furrow planting pattern with maize planted in furrows and wheat planted on ridges combined with straw mulching significantly enhanced summer maize grain yield in the normal year, winter wheat grain yield in the dry year, and annual grain yield in both the dry year and the normal year. All the ridge and furrow planting patterns significantly enhanced the nutrient and protein content in winter wheat grains. EYRF was more conducive to improving grain quality by increasing the P, K, total protein, glutenin and storage protein content in winter wheat grains compared to PRF. However, PRFNR significantly reduced the glutenin content and glutenin/gliadin ratio compared to conventional flat planting, adversely affecting wheat quality. PCA and TOPSIS analysis consistently demonstrated that a ridge and furrow created each year with a border ridge with 133 cm of row space (EYRFBR) was the optimal planting pattern for achieving both a high yield and quality of wheat annually in a dryland maize–wheat double-cropping system. In contrast, a permanent ridge and furrow with normal ridge of 100 cm row space (PRFNR) was more effective for promoting maize yield.

## Figures and Tables

**Table 1:** Effect of precipitation types on grain yield of summer maize, winter wheat, and annual yield in dryland summer maize–winter wheat double-cropping system.

Precipitation Type	Summer Maize	Winter Wheat	Annual
Dry year	4151.1 c	2307.6 b	7621.3 b
Normal year	5446.7 b	3478.4 a	9054.4 a
Rainy year	7605.4 a	3597.4 a	9789.1 a
ANOVA			
Precipitation types (P)	65.1 **	5.4 **	5.2 **
Treatment (T)	4.3 **	0.9 ns	1.9 ns
P × T	0.6 ns	0.2 ns	0.1 ns

Different lowercase letters following the data in the same column indicate a significant difference at *p* < 0.05 level. ** indicate that the variance analysis was significant at the *p* < 0.05 and *p* < 0.01 levels, respectively. ns indicates insignificance.

**Table 2:** Precipitation type in the experiment years from 2015 to 2021.

Year	Summer Maize			Winter Wheat			Annual		
	Precipitation (mm)	*DI*	Precipitation Type	Precipitation (mm)	*DI*	Precipitation Type	Precipitation (mm)	*DI*	Precipitation Type
2015 (2015–2016)	211.3	−1.10	Dry	217.6	0.31	Normal	428.9	−0.72	Dry
2016 (2016–2017)	327.0	−0.16	Normal	218.5	0.32	Normal	545.5	0.02	Normal
2017 (2017–2018)	295.3	−0.42	Dry	322.7	1.98	Rainy	618.0	0.48	Rainy
2018 (2018–2019)	410.8	0.52	Rainy	93.7	−1.67	Dry	504.5	−0.24	Normal
2019 (2019–2020)	437.7	0.74	Rainy	212.0	0.21	Normal	649.7	0.68	Rainy
2020 (2020–2021)	228.1	−0.96	Dry	180.0	−0.3	Normal	408.1	−0.85	Dry

Note: DI represents the drought index. DI = (P − M)/σ, where P is the actual precipitation of the crop growth season or annual year (mm), M is the average precipitation of the crop growth season or annual year (mm), and σ is the mean square deviation of the average precipitation of the crop growth season or annual year. DI > 0.35 is a rainy year, −0.35 < DI < 0.35 is a normal year, and DI < −0.35 is a dry year.

**Table 3:** Treatment description.

Treatment	Field Management Description
PRFBR	Manually ridged and furrowed only in 2004 when the experiment began. The ridge is 95 ± 5 cm wide and 20 cm high. A furrow with a width of 40 ± 5 cm was formed where two ridges met. After the first year, the ridge height was manually restored each year before wheat sowing but never re-ridged. In the summer maize growing season, 1 row of maize was plated in the furrow with 133 cm row space, and previous wheat straw with 0–40 cm height was mulched on the ridge. In the winter wheat growing season, 6 rows of wheat were planted on the ridge with 15 cm space, and 50% of previous maize straw (whole stalk) was mulched in the furrow.
EYRFBR	Manually ridged and furrowed each year, after plowing (20–30 cm) but before wheat sowing. The size of the ridge and furrow and other parameters were same as for PRFBR.
PRFNR	The ridge is 60 ± 5 cm wide and 20 cm high. The furrow is 40 ± 5 cm wide. One row of maize was planted in the furrow with 100 cm row space, and four rows of wheat were planted on the ridge with 15 cm space. The ridging method and other parameters were the same as for PRFBR.
EYRFNR	Manually ridged and furrowed each year, after plowing (20–30 cm) but before wheat sowing. The size of ridge and furrow and other parameters are the same as for PRFNR.
CF	Plowed 25–30 cm before crop sowing in each growth season. Both maize and wheat were planted in a conventional flat cropping pattern and no straw mulching, with row spacing of 80 cm for maize and row spacing of 20 cm for wheat, respectively.

## Data Availability

The original contributions presented in this study are included in the article. Further inquiries can be directed to the corresponding authors.

## Supplementary Materials

The following supporting information can be downloaded at: https://www.mdpi.com/article/10.3390/plants14193030/s1, Table S1: Load value, eigenvalue and contribution rate in principal component analysis.
