# Supplementary material for: Effects of Ridge and Furrow Planting Patterns on Crop Yield and Grain Quality in Dryland Maize–Wheat Double Cropping System
Source: Plants (Basel). 2025 Sep 30;14(19):3030. doi: 10.3390/plants14193030 (PMC12525680; doi:10.3390/plants14193030)
Supplement: Supplementary file 1 [file plants-14-03030-s001.zip › plants-3839093-supplementary.pdf]

Table S1. Load value, eigenvalue and contribution rate in principal component analysis

Index	Principal component load value										
	Summer maize			Winter wheat			Annual				
	PC1	PC2	PC3	PC1	PC2	PC3	PC1	PC2	PC3	PC4	PC5
Summer maize grain yield	0.421	0.898	−0.11				0.257	−0.491	0.557	0.109	−0.554
Summer maize grain protein yield	0.879	0.268	−0.37				−0.177	−0.635	0.255	0.596	−0.239
Summer maize grain protein content	0.591	−0.70	−0.29				−0.523	−0.226	−0.339	0.569	0.345
Summer maize grain P content	0.732	−0.31	0.388				0.095	−0.61	−0.425	0.302	0.296
Summer maize grain K content	0.298	0.126	0.885				0.304	−0.555	−0.439	−0.445	−0.122
Winter wheat grain yield				0.517	−0.44	0.712	0.754	−0.445	0.352	−0.155	0.213
Winter wheat grain protein yield				0.548	−0.403	0.717	0.778	−0.405	0.368	−0.149	0.216
Winter wheat grain protein content				0.738	0.326	0.415	0.739	0.355	0.374	0.152	0.076
Winter wheat grain P content				0.716	0.511	0.035	0.588	0.592	0.136	0.276	−0.273
Winter wheat grain K content				0.622	0.338	0.115	0.517	0.476	0.040	0.314	0.362
Winter wheat grain albumin content				0.947	−0.218	−0.16	0.893	0.070	−0.376	0.147	−0.141
Winter wheat grain globulin content				0.415	0.322	−0.11	0.326	0.425	−0.137	−0.297	0.236
Winter wheat grain gliadin content				0.840	−0.255	−0.46	0.725	0.108	−0.634	0.106	−0.168
Winter wheat grain glutenin content				−0.01	0.963	0.154	−0.140	0.813	0.453	0.055	0.019
Winter wheat grain glutenin/gliadin				−0.56	0.715	0.396	−0.566	0.387	0.677	−0.038	0.126
Winter wheat soluble proteins				0.966	−0.094	−0.17	0.891	0.192	−0.375	0.039	−0.052
Winter wheat storage proteins				0.694	0.612	−0.25	0.486	0.787	−0.142	0.136	−0.124
Annual grain yield							0.664	−0.585	0.426	0.058	0.122
Annual grain protein yield							0.801	−0.243	0.473	0.014	0.186
Eigenvalue	1.925	1.497	1.172	5.521	2.878	1.738	6.689	4.504	3.075	1.373	1.076
Contribution rate (%)	38.490	29.93	23.44	46.00	23.98	14.48	35.207	23.706	16.182	7.227	5.665
Cumulative contribution rate (%)	38.490	68.42	91.87	46.00	69.988	84.47	35.207	58.913	75.094	82.322	87.987
